# Aflatoxin B1 Exposure Suppresses the Migration of Dendritic Cells by Reshaping the Cytoskeleton

**DOI:** 10.3390/ijms26041725

**Published:** 2025-02-18

**Authors:** Kaiyi Tang, Jiaxiong Tian, Yujun Xu, Guofu Shang, Xiaoyan Peng, Ping Yue, Yun Wang, Sen Chen, Zuquan Hu

**Affiliations:** 1 Immune Cells and Antibody Engineering Research Center in University of Guizhou Province, Key Laboratory of Biology and Medical Engineering, School of Biology and Engineering (School of Modern Industry for Health and Medicine)/School of Basic Medical Sciences, Guizhou Medical University, Guiyang 550025, China; tangkaiyi@gmc.edu.cn (K.T.); 2024120111302@stu.gmc.edu.cn (J.T.); xuyujun@stu.gmc.edu.cn (Y.X.); shangguofu@stu.gmc.edu.cn (G.S.); p-yue@gmc.edu.cn (P.Y.); wangyun@gmc.edu.cn (Y.W.); 2 Key Laboratory of Infectious Immune and Antibody Engineering in University of Guizhou Province, Engineering Research Center of Cellular Immunotherapy of Guizhou Province, Guizhou Medical University, Guiyang 550025, China; pengxiaoyan@gmc.edu.cn; 3 Guizhou Key Laboratory of Microbio and Infectious Disease Prevention & Control, Guizhou Medical University, Guiyang 550025, China

**Keywords:** Aflatoxin B1, dendritic cells, cytoskeleton

## Abstract

Exposure to Aflatoxin B1 (AFB1) is considered a significant risk factor for human diseases, including the immune function impairment of immune cells. Dendritic cells (DCs), as essential antigen-presenting cells, play a pivotal role in bridging innate and adaptive immunity. However, the impact of AFB1 exposure on DCs has not been fully elucidated. In this study, we investigated the effects of AFB1 exposure on the migration ability of DCs and its underlying action model. Initially, we observed that AFB1 exposure inhibited the survival of DCs and altered their cellular morphology. Further investigation revealed that AFB1 promotes cell adhesion and inhibits DC migration by modulating the expression of cell adhesion molecules. Additionally, our findings indicated that cytoskeletal remodeling plays a crucial role in these processes. Experimental techniques such as immunofluorescence and RNA sequencing confirmed that AFB1 exposure regulates the expression of cytoskeleton-related genes. Moreover, we found that the perturbation of the gene expression profile through AFB1 exposure is associated with cell communication. Collectively, our study findings demonstrate that AFB1 can disrupt the expression of cytoskeleton- and adhesion-related molecules in DCs, thereby altering cell morphology and migration. These insights could provide new perspectives for further understanding the immunosuppressive effects of AFB1 and developing therapeutic strategies for diseases associated with AFB1 exposure.

## 1. Introduction

Dendritic cells (DCs), as indispensable antigen-presenting cells, bridge innate and adaptive immunity within the immune system [1]. Originating from hematopoietic stem cells, DCs undergo a multifaceted differentiation process, guided by an array of cytokines and growth factors, ultimately differentiating into distinct subtypes with specialized functionalities. Functionally, DCs can be divided into immature DCs (imDCs) and mature DCs (mDCs) differentiation stages [2,3]. ImDCs are primarily located in peripheral tissues, where they are responsible for capturing and processing antigens, playing a crucial role in immune surveillance [4,5]. Upon capturing an antigen, imDCs migrate to secondary lymphoid organs, where they gradually mature into mDCs and present antigen peptides to naive T cells, thereby initiating an immune response [5,6].

The functionality of DCs is intimately tied to their morphology and migratory capacity. The cytoskeleton, comprising actin filaments, microtubules, and intermediate filaments, is indispensable for maintaining cell shape, enabling motility, and facilitating efficient antigen uptake [7,8]. The organization and dynamics of the cytoskeleton not only dictate the mechanical attributes of DCs, such as stiffness and deformability, but also play a determinant role in their ability to migrate to lymphoid tissues for effective antigen presentation [9,10,11]. Furthermore, biophysical characteristics and environmental toxins can further regulate these processes, impacting DC maturation and activation states [11,12,13]. Therefore, elucidating how various factors, including environmental toxins, influence the cytoskeletal organization and mechanical properties of DCs is paramount for gaining insights into their functional roles in immune responses.

Aflatoxin B1 (AFB1) is a potent mycotoxin produced by *Aspergillus* species, notably *A. flavus* and *A. parasiticus* [14]. AFB1 exposure is recognized as a significant risk factor for human diseases, particularly liver cancer, due to its hepatotoxic and carcinogenic properties [14,15,16]. Emerging evidence suggests that AFB1 can also influence immune function [17,18]. AFB1 can induce immunosuppression either through direct toxic effects on the host or by disrupting the immune response, which may subsequently result in secondary infections [19,20]. Numerous studies have documented the immunomodulatory effects of AFB1 and its metabolites. However, the precise mechanisms underlying these effects remain unclear. Given the critical role of dendritic cells in immune surveillance and response, it is vital to explore the impact of AFB1 on DCs.

The aim of this study is to investigate the impact of AFB1 exposure on the migration function of DCs and its underlying mechanism, specifically focusing on its effects on cell viability, morphology, adhesion, Young’s modulus, cytoskeletal dynamics, and gene expression profiles.

## 2. Results

### 2.1. AFB1 Reduces Viability and Alters Morphology of DCs

**Figure 1:**
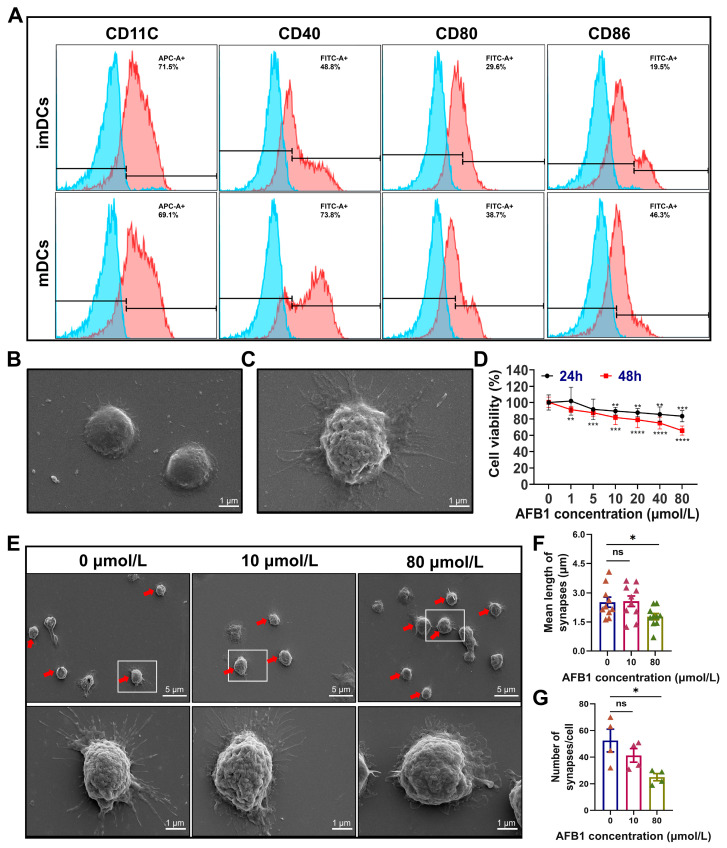
Effects of AFB1 on the survival and morphology of DCs. (**A**) Immunophenotypic molecular identification of DCs. The blue histogram represents unstained control cells, and the red histogram refers to cells stained with the fluorescein-conjugated antibody. APC: allophycocyanin; FITC: fluorescein isothiocyanate; (**B**,**C**) Morphological characteristics of imDCs (**B**) and mDCs (**C**) observed under SEM at a magnification of ×22,000. (**D**) Viability of DCs treated with 0, 1, 5, 10, 20, 40, and 80 µmol/L AFB1 for 24 and 48 h. (**E**) Morphological changes in DCs after AFB1 treatment observed under SEM at magnifications of ×5000 and ×22,000. Living DCs as the red arrows indicated were used for statistical analysis in (**E**), and the representative DCs in the white boxes are enlarged and displayed at lower panel. (**F**) Mean length of synapses of DCs. (**G**) Number of surface synapses on DCs. ns, no significant difference; * *p* < 0.05; ** *p* < 0.01; *** *p* < 0.001; **** *p* < 0.0001.

To investigate the effects of AFB1 on DCs, imDCs were first isolated from the bone marrow of mice. The isolated imDCs were characterized by the detection of DC-specific markers through flow cytometry and by the examination of their morphology using scanning electron microscopy (SEM) (Figure 1A,B). Upon stimulation with lipopolysaccharide (LPS), these imDCs were capable of differentiating into mDCs, as shown in Figure 1C. Subsequently, we treated the imDCs with various concentrations of AFB1 and assessed their cell viability at 24 and 48 h post-treatment. The results indicated that AFB1 significantly reduced the viability of imDCs (Figure 1D). Next, we analyzed the morphological changes in DCs following AFB1 treatment using SEM. From the perspectives of both the number of cellular protrusions and membrane saturation, the cellular morphology of DCs underwent alterations, irrespective of the concentration of AFB1 used (Figure 1E–G). These findings provide evidence that AFB1 not only inhibits the viability of imDCs, but also alters their cellular morphology.

### 2.2. AFB1 Promotes Cellular Adhesion by Regulating Adhesion Molecule Expression in DCs

**Figure 2:**
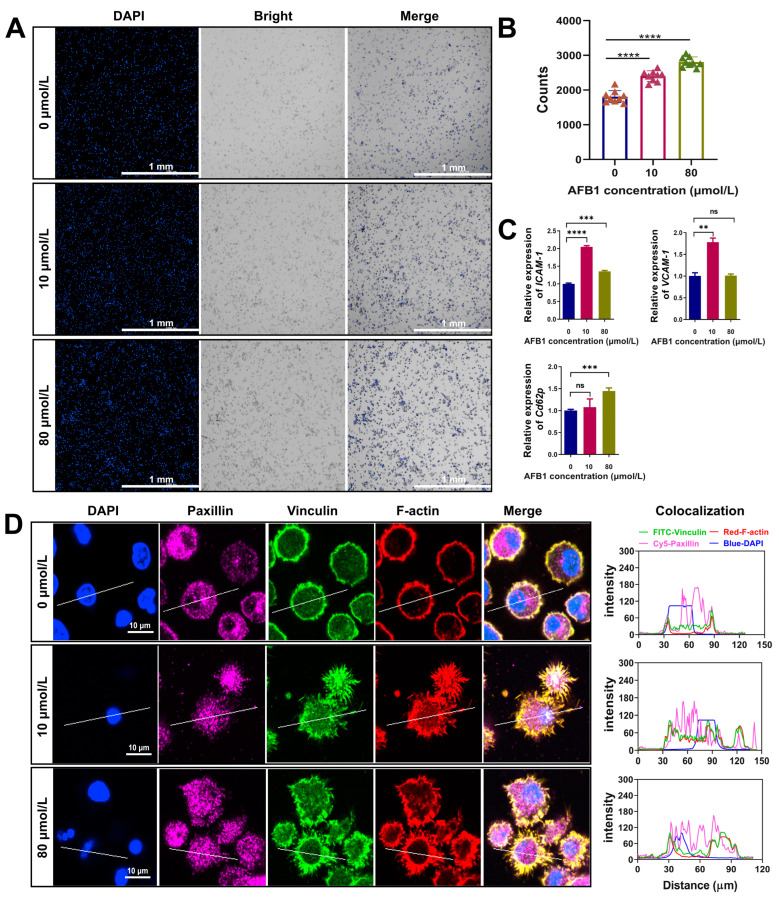
AFB1 enhances cell adhesion by modulating the expression of adhesion molecules in DCs. (**A**) After 24 h of DC adherence, DAPI staining was performed and the number of cells adhering to the well plates was detected by fluorescence microscopy. (**B**) Statistical analysis of the number of adherent cells. (**C**) RT-qPCR analysis of intracellular adhesion molecules. (**D**) Confocal laser scanning microscopy was employed to analyze the expression and subcellular localization of paxillin and vinculin in DCs after AFB1 treatment. DAPI: 4′,6-diamidino-2-phenylindole; Cy5: cyanine 5; FITC: fluorescein isothiocyanate; ns, no significant difference; ** *p* < 0.01; *** *p* < 0.001; **** *p* < 0.0001.

Changes in cell morphology can significantly impact cell adhesion, as alterations in cell shape and structural integrity often affect the function and organization of adhesion molecules, thereby influencing cellular attachment and interaction with the extracellular matrix. We sought to investigate whether AFB1 influences cellular adhesion. Initially, we employed microscopic imaging to assess the number of adhering cells in AFB1-treated DCs. The findings indicated that AFB1 increases the number of adhering cells in a concentration-dependent manner (Figure 2A,B). Subsequently, we conducted Real-Time Quantitative Polymerase Chain Reaction (RT-qPCR) analysis to investigate the expression of adhesion-related genes, revealing that AFB1 is capable of upregulating the expression of *ICAM-1*, *VCAM-1*, and *Cd62p (Selp)* in DCs (Figure 2C). Concordantly, immunofluorescence assays further confirmed that AFB1-treated DCs exhibited an increased expression level of paxillin and vinculin proteins (Figure 2D). In addition, we analyzed the subcellular localization of these proteins. We observed that paxillin in AFB1-treated DCs exhibited a diffuse distribution, with expression detectable in both the nucleus and cytoplasm; in untreated DCs, paxillin was primarily localized to the cytoplasm (Figure 2D). Vinculin was mainly localized to the cytoplasm, but after AFB1 treatment, a small amount of expression became visible in the nucleus (Figure 2D). These observations provide evidence that AFB1 regulates the expression of adhesion-molecule-related genes in DCs and promotes cellular adhesion.

### 2.3. AFB1 Inhibits the Migration of DCs

**Figure 3:**
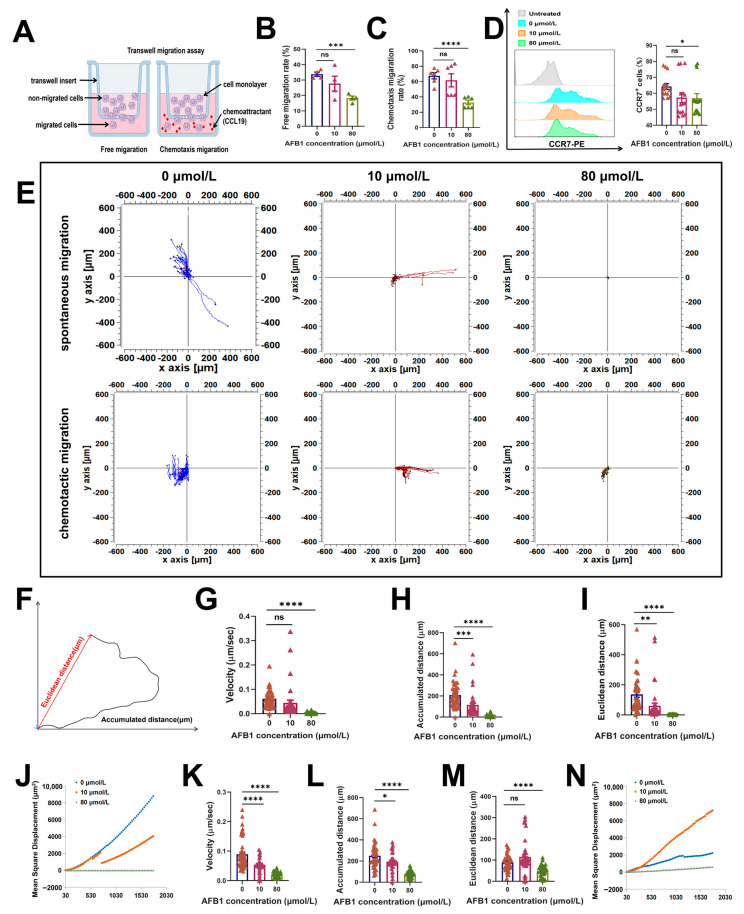
AFB1 inhibits LPS-induced migration of DCs. (**A**) Schematic diagram of the Transwell migration assay. (**B**) Statistical graph of spontaneous migration of DCs. (**C**) Statistical graph of chemotactic migration of DCs. (**D**) Statistical graph of CCR7 expression levels in DCs. PE: phycoerythrin. (**E**) Observation of DC motility using a live-cell imaging station. The aggregated trajectories of DCs over 30 min of spontaneous and chemotactic migration, captured at a frame rate of 30 s per frame. (**F**) Schematic representation of cumulative distance and Euclidean distance traveled by the cells. (**G**–**I**) Analysis of average velocity, cumulative distance, and Euclidean distance of spontaneous migration of DCs using the Chemotaxis and Migration Tool software (v1.01). (**J**) MSD analysis of spontaneous migration of DCs using DiPer software (v1.0). (**K**–**M**) Analysis of average velocity, cumulative distance, and Euclidean distance of chemotactic migration of DCs using Chemotaxis and Migration Tool. (**N**) MSD analysis of chemotactic migration of DCs using DiPer software (v1.0). ns, no significant difference; * *p* < 0.05; ** *p* < 0.01; *** *p* < 0.001; **** *p* < 0.0001.

The adhesive capability of cells exerts an influence on their migratory capacity; accordingly, we postulate that AFB1 additionally modulates the migratory capability of DCs. In order to validate this postulation, we employed the Transwell assay to assess both the spontaneous and chemotactic migration capabilities of DCs (Figure 3A). The results demonstrated a significant decline in both the spontaneous and chemotactic migration abilities of DCs following AFB1 treatment (Figure 3B,C). This finding aligns with the conclusion that AFB1 enhances the adhesion of DCs. CC-chemokine receptor 7 (CCR7), a crucial chemokine receptor regulating DC migration, localization, development, and differentiation, was further examined. Through flow cytometry analysis, we found a significant downregulation of CCR7 expression on the surface of DCs treated with AFB1 (Figure 3D). Furthermore, we employed time-lapse photography for cellular tracking to capture the movement trajectories of DCs inoculated onto a substrate of uniform stiffness over a period of 30 min, following exposure to AFB1 (Figure 3E). The results indicated that the velocity of free migration, migration distance, and mean squared displacement (MSD) of DCs treated with AFB1 on a substrate were significantly reduced (Figure 3G–J). Additionally, the MSD exhibited a power-law relationship with time. Similarly, the chemotaxis migration results also demonstrated that AFB1-treated DCs exhibited slower migration velocities and shorter migration distances (Figure 3K–N). Collectively, these results indicate that AFB1 inhibits the migratory ability of DCs.

### 2.4. AFB1 Alters DC Morphology by Reshaping Cytoskeleton and Decreasing Cell Rigidity

**Figure 4:**
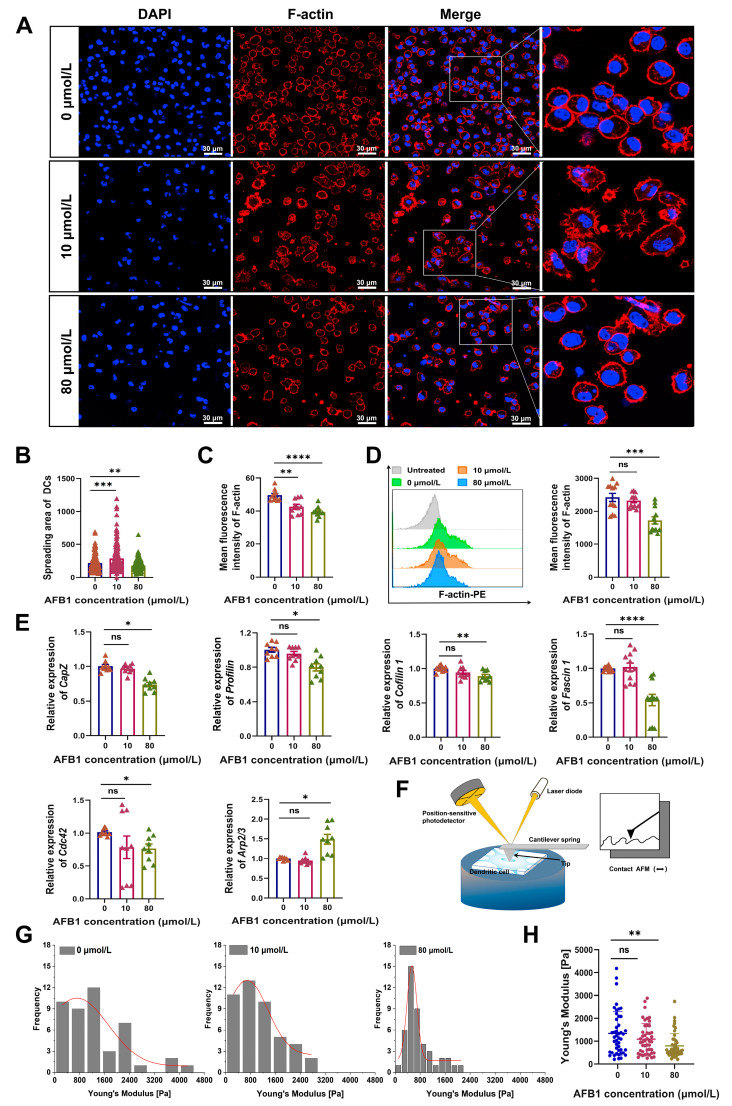
AFB1 alters DC morphology by remodeling the cytoskeleton and reducing cellular stiffness. (**A**) Confocal laser scanning microscopy images showing the distribution of F-actin within DCs. (**B**) Statistical analysis of F-actin fluorescence intensity within DCs. (**C**) Analysis of the spreading area of DC bodies. (**D**) Flow cytometry analysis of F-actin content within DCs. PE: phycoerythrin. (**E**) Expression analysis of genes related to cytoskeleton regulation. (**F**) Schematic diagram illustrating the principle of Atomic Force Microscopy (AFM) for measuring cellular stiffness. (**G**) Histogram of cellular elastic modulus of DCs after AFB1 treatment. (**H**) Statistical plot of Young’s modulus of DCs after AFB1 treatment. ns, no significant difference; * *p* < 0.05; ** *p* < 0.01; *** *p* < 0.001; **** *p* < 0.0001.

The cytoskeleton and focal adhesions constitute the functional underpinnings of cellular migration and adhesion [21,22]. The reorganization of the cellular cytoskeleton represents a pivotal internal mechanism underlying changes in cell morphology [23]. Consequently, we investigated the effects of AFB1 on the cellular cytoskeleton. Through immunofluorescence analysis, we examined DCs exposed to AFB1 and observed that these cells progressively spread, accompanied by a decrease in the average fluorescence intensity of the cytoskeletal protein F-actin (Figure 4A–C). Consistent with these observations, flow cytometry analysis further confirmed a reduction in the average fluorescence intensity of F-actin in AFB1-treated DCs (Figure 4D). These findings suggest the possibility that AFB1 may regulate the expression of genes associated with cytoskeletal structure. To test this hypothesis, we performed RT-qPCR analysis on six genes typically involved in cytoskeletal reorganization: *Profilin*, *CapZ*, *Fascin1*, *Cofilin*, *Cdc42*, and the *Arp2/3 complex*. As expected, while low concentrations of AFB1 did not significantly alter the expression of these six genes in DCs, high concentrations significantly downregulated *Profilin*, *CapZ*, *Fascin1*, *Cofilin*, and *Cdc42*. Conversely, the expression level of the *Arp2/3 complex* was notably upregulated (Figure 4E). Additionally, considering that alterations in the mechanical properties of the cell surface can impact cell morphology, we analyzed the Young’s modulus of DCs after AFB1 treatment. Our results demonstrated a concentration-dependent and significant decrease in the Young’s modulus of AFB1-treated DCs, suggesting that AFB1 treatment diminishes DC rigidity, thereby facilitating morphological changes (Figure 4F–H). In summary, our research reveals that AFB1, through the modulation of cytoskeleton-associated gene expression and the subsequent restructuring of the cytoskeleton, coupled with a reduction in cellular rigidity, ultimately triggers changes in cell morphology. This morphological transformation ultimately influences the cellular adhesive properties and migratory capabilities.

### 2.5. Gene Expression Profiles Perturbed by AFB1 in DCs Are Enriched in Cell Communication and Cytoskeletal-Related Pathways

**Figure 5:**
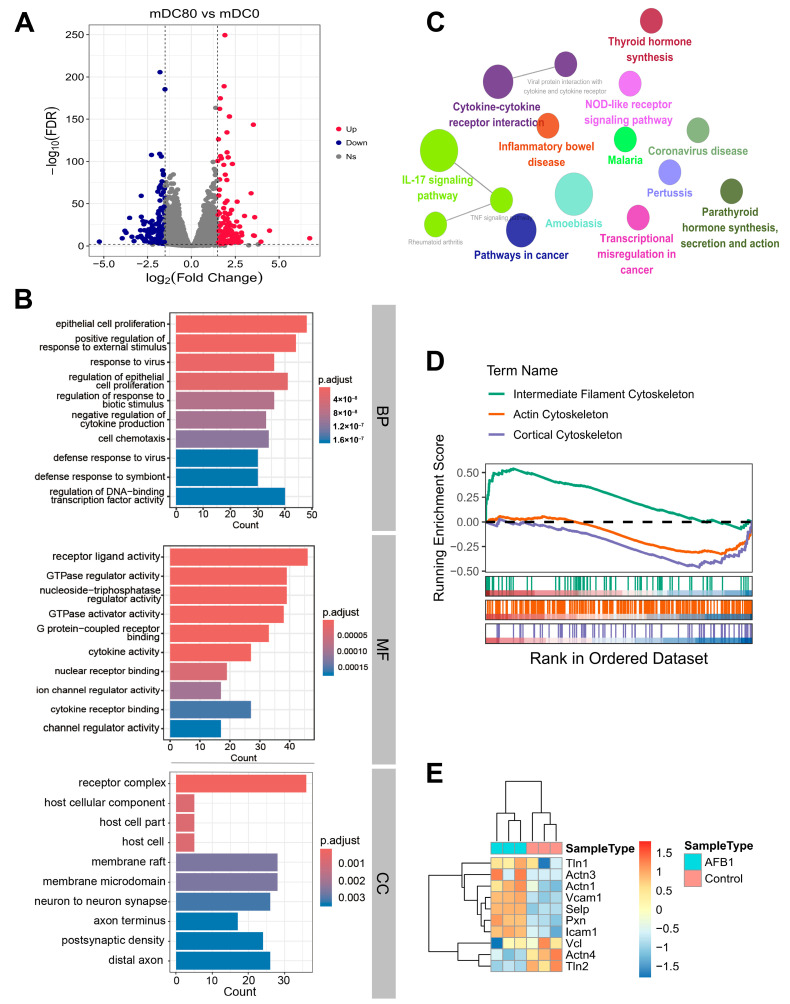
Gene expression profiles perturbed by AFB1 in DCs. (**A**) Volcano plot depicting the differentially expressed genes (DEGs) comparing 80 µmol/L AFB1-treated versus control samples, with genes considered significant at |log_2_FC| ≥ 1.5 and FDR < 0.05. ns, no significant difference. (**B**,**C**) GO enrichment analysis (**B**) and KEGG enrichment analysis (**C**) of the identified DEGs. (**D**) GSEA ranked by log_2_FC. (**E**) Heatmap representing the expression of adhesion-molecule- and cytoskeletal-related genes in DCs based on RNA-seq data, with TPM values normalized to z-scores. Tln: talin-1; Actn: actinin alpha; Vcam-1: vascular cell adhesion molecule 1; Selp: selectin P; Pxn: paxillin; Icam-1: intercellular cell adhesion molecule-1; Vcl: vinculin.

To systematically evaluate the impact of AFB1 on DCs at the gene expression level, we conducted RNA-sequencing (RNA-seq) analysis on both AFB1-treated and untreated DCs. This analysis yielded expression profiles for 18,682 protein-coding genes (Supplementary Table S1). Principal component analysis (PCA) revealed that the AFB1-treated and untreated DCs clustered into two distinct groups (Supplementary Figure S1). Subsequently, we performed differential expression analysis to further investigate these differences. The analysis revealed that 924 genes were significantly differentially expressed in AFB1-treated DCs, with 452 genes upregulated and 472 genes downregulated (Figure 5A; Supplementary Table S2). Gene ontology (GO) enrichment analysis indicated that these differentially expressed genes were significantly enriched in biological processes such as epithelial cell proliferation and the positive regulation of the response to external stimuli (Figure 5B). Molecular function enrichment analysis showed that the functions of these genes were primarily related to cell communication and signal transduction, encompassing receptor ligand activity, cytokine activity, and GTPase regulator activity (Figure 5B). Additionally, these differentially expressed genes were significantly enriched in pathways associated with receptor complexes and membrane microdomains (Figure 5B). Consistent with this, Kyoto Encyclopedia of Genes and Genomes (KEGG) enrichment analysis also revealed significant enrichment of these genes in pathways related to cell communication and signal transduction, including cytokine–cytokine receptor interaction and the IL-17 signaling pathway (Figure 5C). Given the regulatory effects of AFB1 on the cytoskeleton and the limitations associated with differential expression analysis, we conducted gene set enrichment analysis (GSEA) to explore the enrichment of gene expression profiles disrupted by AFB1 within cytoskeleton-related gene sets. The results demonstrated that gene disturbances in AFB1-exposed DCs were significantly enriched in gene sets related to intermediate filaments, the actin cytoskeleton, and the cortical cytoskeleton (Figure 5D). Notably, our transcriptome data also revealed the regulation of the expression of adhesion-molecule- and cytoskeletal-related genes by AFB1. These findings further corroborate the impact of AFB1 on cell morphology through the modulation of the expression of cytoskeletal- and adhesion-molecule-related genes (Figure 5E).

## 3. Discussion

One of the proposed mechanisms underlying the toxicity of AFB1 involves its metabolites causing DNA damage, which subsequently promotes cellular apoptosis [19,24]. Another theory attributes toxicity to oxidative stress, inducing apoptosis, oxidative stress, endoplasmic reticulum stress, and autophagy, ultimately leading to cellular mutations and cancer [25,26,27]. Clinicians have long observed the impact of AFB1 on the immune systems of both humans and animals. Reports have indicated that AFB1 enhances the immunotoxicity in human lymphoblastoid T-cells at the transcriptome level [28]. Furthermore, exposure to AFB1 disrupts intestinal immune function via a mechanism mediated by soluble epoxide hydrolase [17]. Immune dysregulation caused by AFB1 may result in immunosuppression, thereby decreasing resistance to infectious diseases and reducing the efficacy of vaccines and treatments in hosts [29,30]. However, there remain gaps and uncertainties in current research regarding the intricate interplay between the potent carcinogenic mechanisms of AFB1 and its modulation of the immune system. Numerous in vivo and in vitro studies have demonstrated that AFB1 can decrease the viability of various immune cells and impair their functions [19]. In this study, we found that AFB1 treatment significantly reduced the survival rate of DCs, altered their morphological characteristics, and inhibited the elongation and the increase in the number of surface protrusions in DCs. These observations align with previous findings on AFB1′s toxic effects on multiple cell types. Previous studies also found that AFB1 can inhibit the proliferation of human astrocytes and other cells [31]. Our data further confirmed that AFB1 cannot only directly affect DC survival but also change cell morphology, potentially impacting DC antigen-presenting capabilities.

Regarding cell adhesion, we observed that AFB1 can significantly enhance DC adhesion ability and regulate the expression and localization of adhesion-related molecules such as paxillin and vinculin. Paxillin and vinculin are also key cytoskeletal proteins and are especially important in the formation and regulation of integrin-mediated cell-extracellular matrix (ECM) adhesions and cadherin-mediated cell–cell junctions. They can interact with various actin-binding and signaling molecules to coordinate cellular responses to environmental cues [32]. Paxillin is a multi-domain adaptor found at the interface between the plasma membrane and the actin cytoskeleton. When phosphorylated on specific Tyr and Ser residues, it serves an important scaffolding role at focal adhesions by recruiting structural and signaling molecules involved in cell movement and migration [33]. Paxillin can also coordinate the spatiotemporal activation of signaling molecules, including *Cdc42*, *Rac1*, and *RhoA* GTPases, by recruiting guanine nucleotide exchange factors (GEFs), GTPase-activating proteins (GAPs), and G protein-coupled receptor kinase-interacting proteins (GITs) to focal adhesions [33]. In addition, paxillin is an essential player in pathological conditions, including oxidative stress, inflammation, endothelial cell barrier dysfunction, and cancer development and metastasis [34]. Vinculin, identified as a component of focal adhesions and adherens junctions more than 40 years ago, is important for cell–cell and cell–matrix adhesion and participates in the reorganization of the actin cytoskeleton [35,36]. It is connected to the cellular microfilament skeleton and anchors microfilaments to the cell membrane, playing an important role in maintaining cell morphology and regulating cell adhesion, motility, proliferation, and survival. When both paxillin and vinculin proteins are involved in focal adhesion formation and actin dynamics, paxillin primarily functions as an adaptor protein that coordinates signaling events and interacts with a wide range of proteins involved in cell adhesion and motility [37,38]. Correspondingly, vinculin is more structural, focusing on stabilizing the connection between the actin cytoskeleton and adhesion sites [39]. Under mechanical stress, paxillin is involved in dynamic changes in focal adhesion assembly and disassembly, while vinculin is important for strengthening and stabilizing adhesions [39,40]. Together, paxillin and vinculin cooperate in the regulation of focal adhesion dynamics, actin organization, and mechanotransduction, all of which are critical for cell adhesion and migration.

These findings are consistent with existing literature indicating that AFB1 can alter cell adhesion properties by influencing focal adhesion proteins [35]. The ultimate outcome is that AFB1 significantly inhibits the migratory capacity of DCs. It is noteworthy that our study has revealed the ability of AFB1 to upregulate the expression of *ICAM-1*, *VCAM-1*, and *Cd62p* in DCs. However, the upregulation of *Icam-1* and *Vcam-1* does not adhere to a dose-dependent pattern in response to AFB1 treatment. Specifically, the expression of *Icam-1* and *Vcam-1* peaks at 10 µmol/L AFB1 but significantly decreases upon treatment with 80 µmol/L AFB1. This biological effect may not conform to a straightforward dose-dependent paradigm. The influence of AFB1 on DC adhesion-related gene expression may be governed by multiple complex mechanisms, including threshold effects in gene expression and intracellular negative feedback regulation. Therefore, the observed non-dose-dependent expression patterns are likely the result of interactions among these intricate mechanisms. However, it is undeniable that AFB1 does indeed regulate the perturbation in the expression levels of these molecules.

Cellular adhesion capacity impacts its migratory potential. Consequently, AFB1 may impair the directional functionality of DCs, thereby influencing their capacity to migrate to lymph nodes and activate primary immune responses. Indeed, in our study, we observed a reduction in the migratory ability of DCs treated with AFB1. CCR7 is a chemokine receptor that plays a crucial role in the migration of immune cells, especially DCs. It binds to specific chemokines and induces directed cell migration, e.g., inducing directed migration of immune cells to lymph nodes. Cells attracted to chemokines follow signals of increased chemokine concentrations toward migration to the source of the chemokine, such as CCL19 and CCL21, which are expressed in secondary lymphoid organs, such as lymph nodes and spleen [41,42,43]. The interaction between CCR7 and its ligands directs the migration of DCs from peripheral tissues to these lymphoid organs, where they interact with T cells to initiate and regulate immune responses [5,6]. The migration of DCs to lymph nodes is driven by CCR7 [44]. Our research has identified that AFB1 downregulates the expression of CCR7 in DCs, thereby suggesting that AFB1 not only impacts the migration of DCs but may also affect their immune functions.

The cytoskeleton not only maintains cell morphology but also connects with the extracellular structure and extracellular matrix, thereby influencing cell adhesion [45,46]. The reorganization of the actin cytoskeleton is crucial for changes in cell adhesion ability and morphology [46]. The actin exists in cells as either polymerized F-actin or monomeric G-actin, and their dynamic equilibrium through depolymerization and polymerization regulates the structure and function of the actin cytoskeleton, thereby affecting cell morphology and adhesion [47]. Through immunofluorescence staining and flow cytometry analysis, we found that AFB1 significantly decreased F-actin expression and reduced cell stiffness. F-actin, in conjunction with actomyosin, constitutes stress fibers that, through their interaction with focal adhesion sites and cellular junctions, exert a pivotal role in cellular motility, morphology, and morphogenesis [48]. Furthermore, studies have demonstrated that stiffness modulates Piezo1 activity in DCs [49,50]. Activation of Piezo1, in turn, influences DC activation and functionality [49], and exhibits a causal relationship with cellular motility and cytoskeletal reorganization [51]. This research further elucidates that AFB1 regulates the expression of genes associated with cytoskeletal remodeling, such as *Profilin*, *CapZ*, and *Fascin1*. These alterations may disrupt the equilibrium of cytoskeletal proteins, leading to cytoskeletal structural reorganization, which could represent one of the mechanisms underlying AFB1-induced cellular morphological changes.

RNA-seq analysis further revealed the extensive transcriptional effects of AFB1. We found that AFB1 treatment significantly altered DC gene expression, particularly in pathways related to cell communication and signal transduction. Our GO and KEGG enrichment analysis results indicated that AFB1-regulated genes are primarily involved in cytokine activity, intercellular communication, and GTPase regulation, which are crucial biological processes for immune cell function. Additionally, GSEA showed that AFB1-induced gene expression changes were significantly enriched in cytoskeletal-related gene sets, further confirming the role of AFB1 in regulating DC morphology and function by affecting cytoskeletal gene expression. Some studies demonstrated that Rho GTPases are crucial regulators for the dynamic assembly of actin cytoskeleton, playing a pivotal role in the organization and remodeling of dendritic spines [52,53]. Alterations in cell shape in DCs are shown to depend on Rho GTPase activity [54]. Furthermore, in DCs lacking CAV-1, the activation of CCR7-driven GTPase Rac1 is impaired, whereas GTPase Rac1 is known to promote actin protrusion. An increase in GTPase activity is associated with F-actin polymerization [32]. Based on our research, the impact of AFB1 on DC gene expression extends into cell cytoskeletal dynamics and signaling pathways that are integral to immune function, such as the involvement of Rho GTPases.

This study delves into the morphological alterations induced by AFB1 on DCs, elucidates its potential to inhibit DC migration, and suggests profound implications for DC immune function. Given the reported impacts of AFB1 on the immune system [17,28], it is plausible that the effects of AFB1 on DCs and other immune cells may act synergistically, thereby contributing to the induction of immunotoxicity and carcinogenicity. However, it is crucial to note that these findings are derived from experiments conducted under extreme concentrations of AFB1 in laboratory settings. Based on an extensive literature review, we acknowledge that AFB1 concentrations in occupational exposure environments vary considerably depending on the specific job type, working conditions, and protective measures employed [55,56]. AFB1 is naturally occurring, and occasionally, its concentration can be very high, such as in a single moldy peanut. More importantly, AFB1 can be used in biological weapons, where concentrations would be extremely high [57]. Recognizing this, we have studied both a lower concentration of 10 µmol/L and a higher extreme concentration of 80 µmol/L. Nevertheless, this does not diminish the validity and significance of the effects of AFB1 on DCs that we have observed. In totality, we found that AFB1 can alter the morphology, stiffness, adhesiveness, and migratory ability of DCs, potentially having profound impacts on host immune responses. Further exploration of the effects of AFB1 on other functions of immune cells, such as antigen presentation, DCs and T cell interaction, immune activation, and tolerance in animal models, could offer new insights into the development of therapeutic strategies for diseases associated with aflatoxin exposure.

## 4. Materials and Methods

### 4.1. Reagents and Antibodies

Recombinant mouse granulocyte–macrophage colony–stimulating factor (rmGM-CSF), recombinant mouse interleukin-4 (rmIL-4), and murine MIP-3β (CCL19) were obtained from Peprotech (Rocky Hill, CT, USA). LPS was purchased from Sigma Aldrich (Sigma, St. Louis, MO, USA). Fetal bovine serum (FBS) was sourced from Vivacell Biosciences (Shanghai, China). The RPMI 1640 medium and antibiotic–antimycotic solution (penicillin–streptomycin) were acquired from Thermo Fisher Scientific (Gibco, Waltham, MA, USA). Aflatoxin B1 was procured from Pribolab (Qingdao, China). Adhesive slides and cover glasses were purchased from Zytek Lab Equipment Co., Ltd. (Suzhou, China). Reagents, such as 4% paraformaldehyde, bovine serum albumin (BSA), Triton X-100, antifade mounting medium, DAPI, poly-L-lysine, and the cell counting kit-8 (CCK-8), were obtained from Solarbio Science & Technology Co., Ltd. (Beijing, China). Rhodamine phalloidin was sourced from Cytoskeleton Inc. (distributed by AbMole Biosciences, Denver, CO, USA). Antibodies against vinculin, fluorescein isothiocyanate (FITC)-conjugated anti-mouse CD80/CD86/CD40, phycoerythrin (PE)-conjugated anti-mouse CCR7, and allophycocyanin (APC)-conjugated anti-mouse CD11c, as well as TRIzol reagent, were purchased from Thermo-Fisher Scientific (Waltham, MA, USA). The first-strand cDNA synthesis kit was acquired from Tiangen Biotech Co., Ltd. (Beijing, China). The quantitative PCR kit, TB Green Premix Ex *Taq*, was obtained from Takara Biotechnology Co., Ltd. (Dalian, China). The Paxillin antibody and Alexa Fluor647-conjugated secondary antibody were sourced from Abcam Trading (Shanghai) Co., Ltd. (Cambridge, UK).

### 4.2. Preparation of Bone-Marrow-Derived Dendritic Cells (BMDCs)

The preparation of BMDCs adhered to a protocol previously described in our study. Briefly, C57BL/6J mice aged 6 to 8 weeks were euthanized, and their tibias and femurs were used to isolate bone marrow cells. These cells were processed to remove red blood cells using lysis buffer. The remaining cells were then resuspended in the RPMI 1640 medium supplemented with 10% FBS and 1% penicillin–streptomycin. To induce differentiation, the cells were cultured with 20 ng/mL of rmGM-CSF and 10 ng/mL of rmIL-4 for 7 days to obtain imDCs. Subsequently, the imDCs were matured into mDCs by incubating them with 1 μg/mL of LPS for 24 h.

### 4.3. Cell Viability Assay

When LPS was added to induce imDCs maturation, AFB1 with various concentrations (1, 5, 10, 20, 40, and 80 µmol/L) were supplemented for the treatment of cells with two distinct durations: 24 h and 48 h. Following the incubation periods, cell viability was quantitatively determined utilizing the CCK-8 assay. The CCK-8 reagent was added to each well containing the treated DCs according to the manufacturer’s instructions, and the plates were then incubated for an additional period to allow for color development. Subsequently, the absorbance at 450 nm wavelength was measured using a microplate reader.

### 4.4. Cellular Adhesion Assay

Firstly, 1% BSA was subjected to a boiling water bath for 10 min to denature it, and then it was filtered through a 0.22 μm sterile membrane under aseptic conditions for later use. The denatured BSA was added to a 48-well plate for coating for 1 h, followed by two washes with the RPMI1640 medium. Next, the cells were plated in a 48-well plate at a density of 2 × 10^5^ cells per well. The plate was then incubated in a culture incubator for 30 min, and the cells were washed three times with PBS. Subsequently, the cells were fixed with 4% paraformaldehyde at room temperature for 15 min and washed twice with PBS. The nuclei were stained with DAPI at room temperature for 5 min, followed by two additional washes with PBS. Finally, images were captured using a Cytation 5 imaging system (BioTek, Winooski, VT, USA), and the data were analyzed with Image J software (v1.48.0).

### 4.5. Cell Migration Assay

Initially, 600 µL of the complete medium was dispensed into each well of a 24-well cell culture plate. Subsequently, Transwell inserts, equipped with 8.0 µm pore size polycarbonate membranes, were meticulously inserted into the respective wells. A suspension containing 2 × 10^5^ cells was then added to the upper chambers. The plate was subsequently placed in a cell culture incubator to allow for a 24 h period of cell migration. Upon completion of the 24 h migration period, the Transwell inserts were carefully removed, and the lower chambers were gently rinsed with RPMI1640 to eliminate any non-migrated cells. The cell suspension present in the lower chambers was thoroughly mixed, and a 10 µL aliquot of the suspension was aspirated for enumeration using a cell counter. The number of cells that had migrated to the lower chambers was counted, and the migration rate was determined by dividing the number of migrated cells by the initial number of cells added. For the chemotaxis migration experiment, the aforementioned procedure was replicated, with the exception that the lower chambers contained a chemotactic factor, CCL19, at a final concentration of 100 ng/µL.

### 4.6. Real-Time Detection of DC Movement

Initially, a population of 2.5 × 10^5^ DCs, pretreated with AFB1, was seeded onto the matrix gel and allowed to achieve equilibrium within a 37 °C incubator for a duration of 10 min. To evaluate spontaneous cell motility, the samples were placed within a live-cell imaging chamber, which was maintained at a constant temperature of 37 °C and supplemented with 5% CO_2_. The real-time imaging parameters were meticulously configured to capture images at a 30 s interval, utilizing a 10 × objective lens, over a span of 30 min. To monitor chemotactic migration, the chemokine CCL19 was introduced to attain a final concentration of 100 nanograms per microliter (ng/µL), and an identical imaging protocol was subsequently executed. The resultant images were then meticulously processed using Image J software to delineate the movement trajectories of the cells, thereby yielding the X/Y coordinates of each cell at discrete time points. A comprehensive analysis was subsequently conducted using the Chemotaxis and Migration Tool software (v1.01), which allowed for the determination of the average speed, accumulated distance, and Euclidean distance traversed by the cells. Additionally, the Displacement Per software (v1.0) was utilized to calculate the mean squared displacement of the cellular population.

### 4.7. Cell Rigidity Assays

Cells were inoculated on slides and left to stand at 37 °C for 30 min. The Young’s modulus of the DCs was measured by AFM (IPK NanoWizard 4, Bruker, Billerica, MA, USA). Tests were performed in contact mode, and 45 cells were measured for each group. A pyramid probe attached to a cantilever tip was employed to make contact with a single cell. The spring constant of the cantilever was 0.022 N/m. The Young’s modulus of the DCs was determined using force versus distance curves according to the Hertz model.

### 4.8. Morphological Analysis of Cells

The cells were fixed in 4% paraformaldehyde for 4 h. The coverslips were then rinsed twice with PBS and five times with purified water. Dehydration was performed using a series of graded ethanol solutions (30%, 50%, 70%, 80%, 90%, and 100%), with one pass through each concentration for 15 min. After dehydration, the samples were freeze-dried in an ultra-low-temperature freezer at −80 °C for 2 h and then vacuum-dried. The samples were placed in the vacuum chamber of the vacuum coating system, and a low vacuum was extracted, typically for 8 h. After drying, the sample was allowed to warm to room temperature, and then was vented and removed from the system. The samples were then bonded with conductive adhesive, sputter-plated with gold, and observed under a field-emission scanning electron microscope (Phenom Nano G2, Eindhoven, North Brabant, The Netherlands). From each sample, cells that exhibited a healthy appearance, such as a raised shape, structural integrity, and some luster, were selected, which are characteristics that indicate that they were alive at the time of the initial observation. The average length and number of cell surface synapses of these cells were statistically analyzed and plotted.

### 4.9. RT-qPCR

Total RNA was isolated using TRIzol, with its concentration being subsequently determined using a spectrophotometer. Reverse transcription, aimed at synthesizing cDNA, was carried out using the High-Capacity cDNA Reverse Transcription Kit (Tiangen Biotech, Beijing, China). The quantitative real-time PCR reactions were set up in the QuantStudio 3 Real-Time PCR System (Thermo-Fisher Scientific) with a reaction mixture containing the cDNA, target-specific primers (Supplementary Table S3), PowerUp SYBR Green Master Mix (Takara, Japan), and a passive reference dye. The expression levels of the genes of interest were normalized to Gapdh as an endogenous control, and the quantification was performed using the QuantStudio 3 Real-Time PCR System. The comparative Ct method (2^−ΔΔCt^) was applied for data analysis. To ensure statistical reliability, the experiment was independently replicated three times on separate occasions, with each replicate utilizing distinct cDNA preparations derived from the cultures being analyzed. The results from these replicates were averaged to yield a single mean quantity value for each mRNA species.

### 4.10. Immunofluorescence Experiment

The cells were fixed with 4% paraformaldehyde for 10 min. Following this, the cells were washed three times with PBS, with each wash lasting 5 min. Permeabilization was performed using 0.25% Triton X-100 for 10 min. The cells were then blocked with 1% BSA for 1 h. Subsequently, the cells were washed a further three times with PBS, with each wash lasting 5 min. Primary antibody incubation was carried out overnight at 4 °C. After recovering the primary antibody, the cells were washed three times with PBS, with each wash lasting 5 min. A secondary antibody was added, and the cells were incubated at room temperature for 1 h. Following this, the cells were again washed three times with PBS, with each wash lasting 5 min. DAPI staining solution was added, and the cells were incubated at room temperature for 10 min. Finally, the cells were examined and photographed under a fluorescence microscope (Nikon, Tokyo, Japan).

### 4.11. RNA-seq Data Analysis

The sequencing of cDNA libraries was conducted on the Illumina platform by BGI (Shenzhen, China), followed by comprehensive bioinformatics analysis, including read filtering, rRNA and reference genome alignment, and gene abundance quantification, all performed according to standard protocols. PCA was conducted using the *prcomp* function in R software (v4.1.0). Differential expression analysis was then carried out using DESeq2 [58], identifying genes with significant changes (FDR < 0.05, |log2 fold change| ≥ 1.5) between the control and AFB1-treated groups. GO enrichment analysis, and GSEA were performed using the clusterProfiler package (v4.0.2) [59] in R. KEGG enrichment analysis was conducted using the GlueGO tool within the Cytoscape software (v3.10.2) platform.

### 4.12. Statistical Analysis

To assess the relationship between two sets of variables, Welch’s *t*-test was employed. To compare variables across multiple groups, a standard one-way ANOVA was utilized. The threshold for statistical significance was established at *p* < 0.05, with all tests conducted on a bilateral basis. The statistical analyses were conducted using R software or Microsoft Excel (v16.0.16924.20124).

## 5. Conclusions

AFB1 can alter the viability, morphology, stiffness, adhesion, and migration of DCs by regulating the expressions of cytoskeleton- and adhesion-related molecules in DCs. Further analysis of gene expression profiles of DCs to AFB1 exposure reveals the differentially expressed genes are enriched in cell communication and cytoskeleton-related pathways. Thus, the results of this study provide a new perspective on the effects of AFB1 on immune cells, particularly the migration function of DCs. It is of significance for exploring the immunosuppressive effects and carcinogenicity of aflatoxins based on the cellular structure and function of DCs from the interdisciplinary perspective of immunology, biophysics. and cell biology.

## Data Availability

Raw data are available on request from the corresponding author.

## Supplementary Materials

The following supporting information can be downloaded at: https://www.mdpi.com/article/10.3390/ijms26041725/s1.
