# Supplementary material for: Aflatoxin B1 Exposure Suppresses the Migration of Dendritic Cells by Reshaping the Cytoskeleton
Source: Int J Mol Sci. 2025 Feb 18;26(4):1725. doi: 10.3390/ijms26041725 (PMC11854954; doi:10.3390/ijms26041725)
Supplement: Supplementary file 1 [file ijms-26-01725-s001.zip › caption.pdf]

Figure S1: Principal component analysis (PCA) plot; Table S1: RNA-Seq expression data; Table S2: Changes in differential gene expression levels in DCs treated with 80 μmol/L AFB1; Table S3: Target-specific primer sequences
