# Supplementary material for: Aflatoxin B1 Exposure Suppresses the Migration of Dendritic Cells by Reshaping the Cytoskeleton
Source: Int J Mol Sci. 2025 Feb 18;26(4):1725. doi: 10.3390/ijms26041725 (PMC11854954; doi:10.3390/ijms26041725)
Supplement: Supplementary file 1 [file ijms-26-01725-s001.zip › Supplementary Table 3.pdf]

Table S3. Target-specific primer sequences

Gene name	Primer sequence（5’-3’）
Arp2/3	Forward primer：CAACTGTCCAAGTCAGTGAAAG
	Reverse primer：CACCACTTTGTCATGTTTTTGC
CapZ	Forward primer：CTGTGTGAAGATCTCCTGTCAT
	Reverse primer：GTTACTCCACGGTGACCTATAG
Cdc42	Forward primer：CAGACTACGACCGCTAAGTTAT
	Reverse primer：CAGCAGTCTCTGGAGTAATAGG
CD62p	Forward primer：TGGGAGCAAGTGTGATAAGATG
	Reverse primer：GAACTGGCATGTGGATTTGTAG
Cofilin1	Forward primer：CAGAAGAAGTGAAGAAACGCAA
	Reverse primer：AGGTTGCATCATAGAGTGCATA
Fascin1	Forward primer：CTACTTTGACATCGAGTGGTGT
	Reverse primer：CGGTTAATCAGCTTCATGAGGA
GAPDH	Forward primer：ACCACAGTCCATGCCATCAC
	Reverse primer：TCCACCACCCTGTTGCTGTA
ICAM	Forward primer：CTGAAAGATGAGCTCGAGAGTG
	Reverse primer：AAACGAATACACGGTGATGGTA
Profilin	Forward primer：GAAGACCTTCGTTAGCATTACG
	Reverse primer：TGACGGGAGTAAAGGTTACTTC
VCAM1	Forward primer：GACATTTACCCAGTTTACAGGC
	Reverse primer：TGACGGGAGTAAAGGTTACTTC
