# Supplementary figures and images for: Aflatoxin B1 Exposure Suppresses the Migration of Dendritic Cells by Reshaping the Cytoskeleton
Source: Int J Mol Sci. 2025 Feb 18;26(4):1725. doi: 10.3390/ijms26041725 (PMC11854954; doi:10.3390/ijms26041725)

# PCA

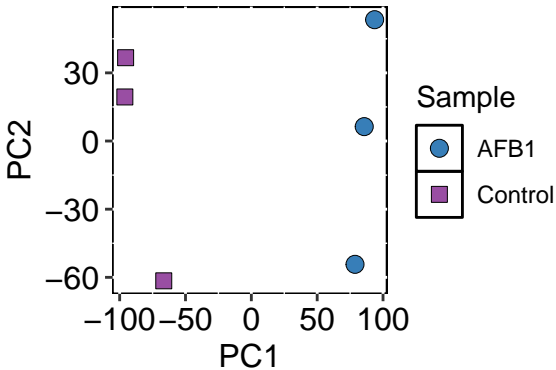

Supplement: Supplementary file 1 [file ijms-26-01725-s001.zip › Supplementary Figure 1.pdf]
